# The misbeliefs and food taboos during pregnancy and early infancy: a pitfall to attaining adequate maternal and child nutrition outcomes among the rural Acholi communities in Northern Uganda

**DOI:** 10.1186/s40795-023-00789-8

**Published:** 2023-11-06

**Authors:** Peter Vivian Acire, Arthur Bagonza, Nicolas Opiri

**Affiliations:** 1 Department of Community Health and Behavioral Sciences, Makerere University School of Public Health, Kampala, Uganda; 2 College of Health Sciences, Makerere University, Kampala, Uganda

**Keywords:** Misbeliefs, Food taboos, Pregnancy, Maternal and child nutrition, Acholi, Northern Uganda

## Abstract

**Background:**

In developing countries, the practice of food taboo is pervasive. The types of foods considered as taboos and the reasons attached to taboos vary from society to society. Food taboos have been recognized as one of the factors contributing to maternal undernutrition in pregnancy, especially in rural settings. In the rural Acholi community where malnutrition is prevalent, very little is known about these food taboos and misbeliefs. This study, therefore, aims to explore various misbeliefs and food taboos in the time of pregnancy that can influence maternal and child nutrition outcomes in Acholi.

**Methods:**

A community-based qualitative cross-sectional study was conducted between April and May 2022 in five districts in the Acholi subregion. Focus group discussions (FGDs) and key informant interviews (KIIs) were used to collect data. Data transcription was done verbatim, organised into themes, assigned unique color codes, and manually analysed thematically.

**Results:**

Upon scrutiny of the transcripts, three themes were eminent. The first theme focused on foods that are considered taboos in Acholi community and the reasons linked to them. Participants indicated offals, chicken, wild birds, smoked meat and fish, sugarcane, garden egg (‘*Tula*’), groundnut, bush meat, mushrooms, honey, sour fruits, or meals (oranges, mango, passion fruits, lemon, tamarind, ‘*Malakwang*’), goat’s meat, ‘*Lalaa*’ (the bitter green leafy vegetable), and ‘*Lamola*’ (*Hyptis spicigera*) as the major taboo foods. The second theme was the reasons underlying the adherence to the food taboos and misconceptions. Cultural dictates, individual characteristics, and societal context were the main reasons for the adherence to food taboos. The third theme looked at the misconceptions and other taboos during pregnancy. It was found that pregnant women are not allowed to touch grave soil, shave their hair, walk over an anthill, slaughter chicken or birds, have sex during pregnancy, sit on animal’s hide or skin, and/or touch needles.

**Conclusions:**

Nutritional counseling and education should focus more on addressing food taboos. The mode of delivery of the nutrition message should be inclusive, targeting pregnant women and their spouses, school-going children, adolescent girls, and cultural leaders at their respective points of contact.

**Supplementary Information:**

The online version contains supplementary material available at 10.1186/s40795-023-00789-8.

## Introduction

Food taboos exist in one form or another in every society on Earth. Nowhere in the world do people, a tribe, or an ethnic group exploit the full potential of edible resources in their surroundings [1]. For example, the hunters and gatherers of the Paraguayan jungle (the Ache people) are believed to exploit only 50 of the several hundred edible mammalian, avian, reptilian, amphibian, and piscine species found in the tropical forest. As with plants, fruits, and insects, only 40 of them are exploited. 98% of the calories in the diet of the Ache are supplied by only seventeen different food sources in the tropical forest [2]. The term food taboo is used to describe the deliberate avoidance of a specific food item for reasons other than a simple dislike of food preferences [3]. However, in almost all societies, food taboos are understood as a systematic set of rules about which foods or combinations of foods may not be consumed [4].

Although ordinary restraint from certain foods may in itself not mean food taboo, regular avoidance usually culminates into a tradition and eventually as a food taboo [2, 5, 6]. Social anthropological research on eating and food taboos underpins habitual avoidance of foods as stemming from utilitarian doctrine and magico-religious motives [5, 7]. The delineation of food taboos as mechanisms for conserving resources as well as a person’s health has been documented by functionalists [8]. The explanation of rituals and taboos based on spiritual, religious, and magic ideation has also been cited [5, 9, 10].

In developing countries, the practice of food taboos is prevalent. The type of food considered taboo and the reasons attached to the taboos vary from society to society. In most settings, food taboos often target pregnant women to prevent what is perceived as harmful effects of these foods on the newborn [11, 12]. For example, a study in South Africa revealed that foods such as meat, fish, potatoes, fruits, beans, eggs, butternut, and pumpkin, which are good sources of essential nutrients, are avoided [13]. In Ethiopia, food items such as dairy products (milk, yogurt, cheese), vegetables, egg, linseed, fruits, sugarcane, honey, and fatty meat are avoided [14]. In Gambia, Nigeria, Gabon, the Democratic Republic of Congo, and Asia, pregnant women are usually forbidden from consuming the richest food sources of iron, carbohydrates, animal proteins, and micronutrients [11, 12, 15].

For many cultures in Uganda, there is a belief that being loyal to food-based taboos often results in a healthy pregnancy. For instance, in Buganda, taboos restrict pregnant women from eating slaughtered animals. In Karamoja, women are debarred from eating the meat of dead animals, offal, and chicken. Lack of adherence to these taboos is believed to result in preeclampsia, general body weakness, being sick, abnormal fetus movement, miscarriages, abdominal pains, malaria and fevers, weight loss, being pregnant beyond the gestation period of nine months, bleeding, vaginal discharge, and sexually transmitted diseases [16, 17]. Other studies elsewhere in the world other than in Uganda also show similar reasons for avoidance of other food items. The avoidance of certain foods was linked to adverse pregnancy outcomes (macrosomia, dystocia, fear of miscarriages or abortion, and fetal abnormalities), labor, and avoiding an undesirable body appearance for the baby [13, 18].

Malnutrition, especially among pregnant women and adolescents, increases the risk of maternal and neonatal mortality and morbidity, low birth weight babies, slow growth, and impaired cognitive development, which often translate to an intergenerational cycle of malnutrition. Addressing existing barriers is critical to ensuring adequate maternal nutrition during pregnancy and lactation. Although adequate dietary intake during pregnancy could be affected by many factors, including food affordability and accessibility, food taboos have been recognized as one of the factors contributing to maternal undernutrition in pregnancy, especially in rural settings [19, 20]. Despite this recognition, little is known about these misbeliefs and food taboos and their contributions to maternal and child nutrition outcomes in Uganda and, in particular, the Acholi subregion due to limited literature.

The Maternal, Infant, Young Child, and Adolescent Nutrition (MIYCAN) 2020 action plan and the National Nutrition Action Plan (UNAP II) of the government of Uganda call for a reduction in all forms of malnutrition among the vulnerable population. However, addressing the growing concerns on maternal child undernutrition will likely require further actions in addition to what is previously envisaged. According to the World Health Organization’s global nutrition targets (2025), Uganda is still off-track in achieving two out of the six critical indicators of a 40% reduction in the number of stunted children and a 50% reduction in anaemia prevalence among women of reproductive age. The Uganda Demographic and Health Survey (UDHS, 2016) shows that one-third of children under five are stunted, with those in rural areas more likely to be stunted (30%) than those in urban settings (24%). Similar to stunting, the prevalence of anaemia is higher in rural areas (54%) than in urban areas (48%). Over half (53%) of the children 6–59 months of age and 32% of the women 15–49 years old are anaemic. However, there is regional variation in the prevalence of anaemia; 71% of children and 47% of women in the Acholi region are anaemic, which is higher than the national average and twofold-fold higher than the prevalence in other regions. The real causes of these marked regional variations remain poorly understood. There are indications that the challenges of malnutrition go beyond physical access to food. Cultural shortcomings seem to play a key role in exacerbating the triple burden of malnutrition in this country.

The Acholi districts in Northern Uganda have been referred to as the breadbasket of Uganda (Remigio, 2010). Despite the abundance of food sources in Acholi, the prevalence of malnutrition, especially anaemia, is still high in children and women. Anecdotal evidence regarding misbeliefs and food taboos among the Acholi communities is eminent and has been in practice for years. Notwithstanding, there is no available literature to fully show and describe the practices or beliefs related to food taboos due to a scanty study in the area. It is likely that the practice of various food taboos among the Acholi communities could be the major reason for poor maternal and child nutrition outcomes in the region and thus could explain the regional variance in nutrition outcomes in the country. This study, therefore, aims to explore various misbeliefs and food taboos in the time of pregnancy that could influence maternal and child nutrition outcomes in Acholi.

## Research methods

### Study setting

This study was conducted in the Acholi subregion located in northern Uganda. The Acholi subregion is composed of eight districts, namely Agago, Amuru, Gulu, Kitgum, Lamwo, Nwoya, Omoro, and Pader. Five districts, Gulu, Omoro, Amuru, Pader, and Kitgum, were sampled for this study. The Acholi, with a population of over 1.5 million, constitutes one of the about 65 ethnic groups in Uganda [21]. The Acholi area covers a greater majority of mainland northern Uganda, occupying approximately 11,264 square miles with the border of South Sudan [22]. This accounts for 12% of the total land area of Uganda. The areas within which the Acholi lives are vastly forested, grasslands, semi dry lands, and swampy. The availability of vast fertile land encourages wildlife hunting, agrotourism, animal rearing, and the growing of various food and cash crops. The main food crops grown in the area are rice, sweet potatoes, sorghum, maize, millet, beans, cassava, and groundnuts, while cotton, tobacco, soybean, and sugarcane, are majorly for income [23, 24]. Livestock such as cattle, small ruminants (e.g., goats, sheep, rabbits), and poultry are reared for both domestic and economic purposes. There are 132 government health facilities in the Acholi subregion. These comprised 116 HC III, 9 HC IV, and 7 hospitals. Depending on the level, these facilities provide outpatient and inpatient services and emergency and obstetric care services [25].

### Research design and participants

This study was conducted between April and May 2022. A community-based qualitative cross-sectional study design using focus group discussions (FGDs) and in-depth interviews (IDIs) of key informants (KIs) was used. The methods were chosen to allow for in-depth probes due to sensitivity around the practices. Participants were selected from five randomly sampled districts, i.e., three from West Acholi Gulu, Omoro, and Amuru, and two others from East Acholi, i.e., Pader and Kitgum. Study participants included the health care providers (HCPs) attached to the antenatal clinic (ANCs), village health teams (VHTs), traditional birth attendants (TBAs), 'Rwodi of Ker Kal Kwaro Acholi’, pregnant women, and their spouses.

### Participant recruitment

Purposive sampling techniques were used to select KI and FGD participants. Pregnant women attending antenatal clinics in five rural government health facilities in the sampled districts, namely, Gulu, Amuru, Omoro, Pader, and Kitgum, were purposively selected to participate in the study. Atiak HC IV in Amuru, Awach HC IV in Gulu, Lalogi HC IV in Omoro, Pajule HC IV in Pader, and Namukora HC IV in Kitgum district were the health facilities purposefully sampled for the study. These facilities were chosen because of their rural location and the capacity to offer obstetric care services. Records of women were obtained from registers available at the health centres. Only those who met the selection criteria (i.e., were pregnant, able to speak the local language, lived in a rural area, were not weak/sick, had a partner living together, and were willing to give consent) were selected. Partners of women attending ANCs automatically became potential KII participants. Phone numbers of spouses of women attending ANC were obtained from the medical records for telephone interviews. The ‘Rwodi me Ker Kal Kwaro Acholi’ (the chiefs of the Acholi chiefdom) were identified and selected from the 54 chiefs listed in ‘Ker Kal Kwaro Acholi’s Strategic Plan 2009 document. Telephone contacts of the ‘Rwodi’ were obtained at the time of selection to schedule interview appointments. Village health teams (VHTs) were identified from the staff inventory available at the targeted health facilities. Lists of the former TBAs registered with the respective health authorities were obtained, and participants were identified to participate in the study.

### Data collection tools and methods

Forty semistructured interviews using a pretested interview guide were conducted with the key informants to gather a range of views regarding misbeliefs and food taboos during pregnancy and early infancy. This data collection method was chosen because it’s appropriate for the research questions being explored. The interview guides used (supplementary file 1) were specifically developed by the researcher for this study. After the development of the tool, it was first piloted with 10 participants who were later excluded from the main study. Questions that showed deviations, or were unclear, too long, and difficult to understand were effectively validated to improve the validity of the tool. Additionally, the interview guides were updated whenever an information gap was discovered during the data collection. In-depth interviews were conducted with the KIs participants, i.e., 10 ‘Rwodi of Ker Kal Kwaro Acholi’’ (the chiefs of the Acholi chiefdom) from across the 5 selected Acholi districts to harness a range of views and opinions, 10 health care providers attached to the Ante-Natal Clinic, and 05 Village Health Team (VHTs), 05 spouses (husbands) of the pregnant women, and 10 former Traditional Birth Attendants (TBAs). Nine FGDs were conducted with purposively selected pregnant women. Interviews with the health care providers and pregnant women were conducted at the health facilities. All the other participants (Rwodi, VHTs, TBAs, and partners of pregnant women) were interviewed from their respective homes either via telephone or face to face. To ensure the reliability of the data being collected, all the interviews were conducted in the medium of Acholi language by the principal investigator (PI) and trained research assistants. The decision on the actual number of in-depth interviews to conduct with the KIs and FGDs with pregnant women was reached based on the level of information saturation, which was ascertained by transcribing the discussions of each day’s session. All data were audio-recorded. Notes and memos of participants’ behavior and contextual aspects were taken during the interviews. The FGDs took on average between 40 and 60 min, while IDIs with the KI took an estimated 30–40 min. Some KI participants, especially ‘Rwodi (the chiefs)’ and TBAs whom the research team could not meet in person, were interviewed on the phone. Additionally, the interviewing of spouses of pregnant mothers was done via telephone calls. Upon completion of data collection, data was checked for errors and inconsistencies to ensure data quality.

### Data analyses

This study employed appropriate statistical analyses method for the research questions and data collected. The notes and voice recordings taken during the focus group discussions and key informant interviews were synthesised verbatim and the principles of systemic text condensation were applied to manually analysed the data [26]. Transcripts were checked several times to fully understand its content, pick out the actual meaning, summarise the content using color coding, and then categorise the main themes based on the condensed text. The data were then thematised. Parts of the discussions were restated verbatim, and some were amended to improve clarity. The results were narratively presented using the verbatim of the respondents to elucidate and provide further proof for key statements. All quotes expressed in the local language (Acholi) were translated into English. Basically, the phases of the thematic analysis of the qualitative data were data transcription, data familiarization, initial code generation, thematic search and reviews, thematic definition and naming, and reporting.

## Results

### Sociodemographic characteristics of the study participants

Nine FGDs were held. Key informant interviews were conducted with 32 participants. Altogether, a total of 118 respondents participated in the nine FGDs (8–10 members for each discussion) and 32 KIIs. The final number of in-depth interviews and FGDs conducted was established based on the level of information saturation, which was ascertained after transcribing the discussions of each day’s session by the research team.

The 118 study participants consisted of 86 pregnant women, 8 Rwodi of Ker Kal Kwaro Acholi, 7 health care providers, 9 former traditional birth attendants, 4 village health teams, and 4 spouses of pregnant women. The participants’ age groups ranged from 16 to 82 years old. Their educational status varied widely, i.e., ranging from no education to tertiary formal education. 95% of participants lived in rural areas, and 83.1% engaged in agriculture as their major occupation, except for health care providers and some ‘Rwodi’ (Table 1).

**Table 1:** Sociodemographic characteristics of respondents

Respondent’s characteristics	Category	Number	Percentage
Age range	15–18	22	18.6
	19–35	55	46.6
	36–45	24	20.3
	46 and above	17	14.4
Sex	Male	17	14.4
	Female	101	85.6
Residence	Rural	112	94.9
	Urban	6	5.1
Occupation	Agriculture	98	83.1
	Formal employment	6	5.1
	Unemployed	14	11.9
Education	No education	35	29.7
	Primary	69	58.5
	Secondary	8	6.8
	Tartiary	6	5.1

Agriculture also includes the informal employment category

Following rigorous reviews and in-depth analysis of the scripts, five key themes were identified:-

- Main factors that determine food choices in the community.
- Foods that are considered special to pregnant women and newborn babies.
- Foods that are considered taboos in the Acholi community and the reasons linked to them.
- Reasons underlying adherence to food taboos and misconceptions.
- Misconceptions and other taboos during pregnancy in the Acholi community.

### Main factors that determine food choices in the community

Participants were asked about their opinions regarding factors that determined food choices during pregnancy in their communities. Varied views were expressed to explain why some people chose what to eat and what not to eat. Most of the respondents, particularly pregnant women, TBAs, and health care providers, believed that most women have specific food preferences because of the existing medical conditions, cultural beliefs, level of income, availability of food items, prescribed menu, individual preference, state of pregnancy, and knowledge about food and nutrition. A KII participant, a traditional birth attendant, stated,

“……. for me I have ulcers and my doctor advised me not to consume sour food such as oranges, *Malakwang*, soda, and or eat beans…….”

Another TBA elaborated on how a household or individual’s economic status can influence which food to eat and what food to forgo.

*“……. If you do not have money, there is no way you can eat the foods you like. Currently, with the current economic crisis and food insecurity, foods have become very expensive and scarce. You just have to eat whatever is available…….”*

One KII participant added,

*“…. the Acholi culture dictates what pregnant women should eat and what they shouldn’t. For example, women are banned from eating bush meat when they are pregnant*. *Thiskind of belief* does *not only restricts women from eating such food but also affects the entire household’s food choices.…….”*

An FGD participant pregnant woman indicated,

*“…… The pregnancy period can be a very confusing moment in a woman’s life, especially in the first and second trimesters. I almost detested all foods in my first three months of pregnancy with my second born. Most often, a simple smell of certain food would make me vomit everything from my stomach….”*

Another KII participant, a health care provider, associated food preference with the education of the woman. She explained that knowledge about nutrition contributes greatly to determining what one should eat.

*“……. If you know the food groups that are good for your health, for example, when you are sick or pregnant, you will rightly choose the types of food to include on the menu…….”*

Similarly, another FGD participant pregnant woman reckoned,

*“……. Food availability is a major reason for differing food choices. You might have cash, but if the food you want to buy is not available in the local market, what can you do? You will have to change your plan and look for alternatives. For me, if I want to eat fish and I don’t find it in the market, I go for silverfish….”*

### Foods that are considered special to pregnant women and newborn babies

Pregnant women and their spouses, healthcare providers, TBAs, and VHTs believed that some foods are ‘special’ for expectant mothers and their newborns. For the pregnant mothers, foods such as meat; leafy vegetables (pumpkin leaves, *boo*, cabbage, *Malakwang*, *Akeyo*); silverfish; millet food; soybean porridge; carrots; milk; fruits (pawpaw; Avocado); and pumpkin leaves were reported as special and should be consumed as much as possible. These foods are perceived to fatten and offer general nourishment to the baby while in the womb. *Malakwang* is consumed specifically to enhance milk let-down during breastfeeding.

For newborns, breast milk is given exclusively in the first two months. Some mothers, however, introduce sugar solution, black tea, and soup immediately after birth. From 3 months onwards, the infants are given soybean, millet, and maize flour porridge mixed with sesame or groundnut paste (peanut butter).

A pregnant mother, a participant of an FGD, indicated,

*“……. when my third born was in the womb, I ate a lot of pumpkin leaves, and the baby came out very big that I almost failed to deliver normally…….”*

A KII participant, one of the traditional birth attendants, also expressed her experience with her first-born son. She stated,

*“……*.*Pasted foodsare miracle meals during pregnancy. In my first pregnancy, my mother-in-law advised me to include pasted beans,Malakwang*, *Boo*, *Akeyo*, *porridge, and meat in my diet. I did that, and the outcome was amazing when I gave birth. My son came out all glittering and very big…….”*

Additionally, another KII participant, a health care provider, stressed that maternal nutrition during pregnancy should be given careful attention. Meal planning should include the different food groups available within the community. These foods should be able to provide essential nutrients for nourishing both the mother and the growing fetus. Thus, foods rich in carbohydrates, proteins, minerals, and vitamins should be included in the daily diet. Micronutrient supplementation, especially of iron and folic acid, is equally important. These can be started immediately before the woman conceives and continued during pregnancy until 3 months postpartum. For infants younger than 6 months, exclusive breastfeeding is advised. The participant added,

*“……. We often advise women when they come for their antenatal visit to eat a balanced diet. Those who cannot afford certain foods can opt for alternatives; for example, if fish are expensive, we advise them to buy silverfish. Those who have land can establish a small kitchen garden to grow vegetables. We also give these women combined iron and folic acid tablets and encourage them to continue intake throughout their pregnancy and after they have given birth. Our routine advice to mothers regarding infant feeding is that infants below 6 months should not be given solid foods. The child should be fed entirely on breast milk except for syrups and vitamins. Breastfeeding should continue until the baby reaches two years….”*

### Foods that are considered taboos in the Acholi community and the reasons linked to them

FGD and KII participants indicated that certain foods are taboos in some Acholi clans and should not be eaten or even touched by pregnant women. Overall, 14 foods are held as taboos across the Acholi community for various reasons (Table 2). FGD and KII participants mentioned avoidance of high carbohydrate foods, especially sugarcane and honey, during pregnancy. Consumption of these foods was viewed to be associated with generalized fetal skin fissures and having a newborn with excessive birth weight, which was believed to lead to a difficult birth. A KII participant, a TBA, stated,

*“……. My son almost killed me if I was not an experienced TBA. He was too big because I ate too much honey in the last three months of my pregnancy. I got a lot of tears during delivery….”*

**Table 2:** Summary of foods considered taboos during pregnancy and reasons attached to them, Acholi subregion, Northern Uganda, 2022

Taboo foods	Reasons for the ban	Participants who mentioned the taboo foods and reasons
Offals	It causes the umbilical cord to tie around the neck of the fetus	Majority of Pregnant women and Health Care Providers
Chicken and chicken’s skin	It causes miscarriage and the skin chokes the fetus	Majority of the TBAs, Pregnant mothers and their spouses
Wild birds	Causes blindness to the fetus	Few pregnant women
Smoked meat and fish	Causes navel/umbilical cord infection	Majority of the pregnant mothers
Sugarcane	Causes generalized fetal skin fissure	Majority of the pregnant mothers, TBAs, and VHTs
Garden egg (*‘Tula’*)	Skin inflamation to the fetus	Most of the TBAs
Groundnut	It causes whitish plasters onto the fetal baby	Majority of Pregnant women
Bush meat	Causes miscarriage	Most of the Rwodi and TBAs
Mushrooms	It causes miscarriage; infertility in women; and body sores to the fetus	Most of the Rwodi and TBAs
Honey	It causes macrosomia	Few of the TBAs
Sour fruits or meals	Causes fetal skin rashes	Few of the TBAs
Goats meat	To show respect for the men	Few of the TBAs, Rwodi and Spouses of pregnant women
*‘Lalaa’* (the bitter green leafy vegetable)	Causes erectile dysfunction (impotence) to the newborn child	Majority of the TBAs, Pregnant mothers and their spouses
*‘Lamola’* (*Hyptis spicigera*) also known as the black sesame	Causes fetal blindness	Majority of the TBAs and Pregnant mothers

Local names of taboo foods are presented in quotes

Similarly, an FGD participant, a pregnant mother, added,

*“…My own brother’s son was born with generalized skin cracks and tears when the mother consumed sugarcane during her pregnancy. It took some time for the wounds to completely heal. In fact, the boy bled too much at the time of his birth that we thought he would die…….“*

The consumption of protein-rich and nutrient-dense foods such as offal, smoked meat, fish, chicken, goat meat, and wild meat is restricted during pregnancy for various reasons depending on the clan. Many of the participants believed that eating offal and chicken skin, particularly during advanced pregnancy, causes ‘baby strangling’, a condition in which the umbilical cord ties around the baby’s neck, leading to choking. Handling or eating smoked meat and fish a few weeks prior to childbirth is feared to cause infection of the umbilical cord after the baby is born. The perception behind barring women from eating goat meat and chicken is basically to show respect for the men. One of the KII participants, Rwot, emphasized,

*“………In Acholi, women are banned from eating goat and chicken because these foods are very small whenever cooked and so served only to the men. Usually, more than one meal is prepared on the day a chicken or goat is cooked. The women eat the other foods while the meat is left for the men. This is a sign of respect that has been in practice from time immemorial…….”*

Likewise, an FGD participant, a pregnant woman, commented,

*“……… My neighbor ate goat’s offals in her pregnancy and her baby came out with the umbilical cord wrapped around the baby’s neck. On the day of her delivery, her husband had also bought some smoked meat. A few days later, the baby developed a serious infection on the umbilical cord stump…….”*

Participants also reported that foods such as fruits, particularly oranges, lemons, passion fruits, mango, and tamarind, including some types of vegetables such as garden eggs (*tula*), ‘*Lalaa*’ (the bitter green leafy vegetable), and ‘*Lamola*’ (*Hyptis spicigera*), are considered taboo for women throughout the gestation period. When probed, participants disclosed various reasons attached to the ban on the consumption of these foods. The garden egg, which some people call the bitter eggplant and in the local language it is referred to as ‘*Tula’*, is known by some communities in Acholi to cause inflammation of the skin of the growing fetus. *Lamola* (*Hyptis spicigera*) is believed to cause fetal blindness, and consumption of ‘*Lalaa’* is thought to cause impotence to the newborn. A few of the KII participants, TBAs, cited eating sour fruits and/or other foods to cause generalized fetal skin rashes. In a discussion to explain this, a Traditional Birth Attendant, KII participant affirmed that,

*“……. it’s forbidden for a woman in my clan to consume anything sour during her pregnancy. Sour foods make the body of the baby look rough and unpleasant…….”*

In the same tone, another TBA, KII participant, stated,

*“……. If you want to lose the sight of your baby just eat ‘Lamola’ when you are pregnant. It happened to my aunt’s daughter; she was born blind. At first, no one knew what had happened to the eyes, but this was confirmed when the mother disclosed she ate ‘Lamola’ when the baby was still in the womb…….”*

Many FGD and KII participants, particularly TBAs, Rwodi, and pregnant women, associated the consumption of raw or roasted groundnuts with the whitish plastered substance that normally appears on the baby immediately after delivery. On the other hand, certain types of wild edible mushrooms are believed to cause miscarriage, infertility in women, and fetal body sores. One of the Rwodi of Ker Kal Kwaro Acholi said,

*“…… A ban on the consumption of mushrooms during pregnancy is unfamiliar in some clans in Acholi. This taboo is common to some specific clans. In some clan, the belief is so strong that when a woman consumes mushroom during pregnancy, she loses the fetus instantly or the mother never ever give birth again…….”*

In contrast, a pregnant woman one of the FGD participants testified,

*“……. I ate lots of raw and roasted groundnuts when I was pregnant with my 2-year-old son, but he came out clean on the day of his delivery. I think some things are simply misconceptions that should be abolished in this generation…….”*

### Reasons that underlie adherence to food taboos and misbeliefs

Based on the views expressed and submissions made by many of the respondents, the reasons that underlie adherence to food taboos can broadly be categorized into three categories, namely, cultural dictates, individual characteristics, and societal context.

### Cultural dictates

It is apparently clear that every clan in Acholi has its own cultural beliefs that they hold so dearly, and it is expected that every member of the clan adheres to them. Failure to comply with the taboos tantamount to being thrown out as an outcast or facing grave consequences. When a man marries a woman, she is first allowed to stay with her mother-in-law for about a year before moving to her own house to start living independently. During this time, she is taken through the dos and don’ts of that family that she must follow. This kind of informal teaching continues for generations after generations. The message about taboo foods is usually passed to children from an informal setting called ‘*wang oo*’ (literally meaning the fireplace). One KII participant, a traditional birth attendant, testified,

*“……. when I eloped, I first stayed with my mother-in-law for eight months. While I was with her, she groomed me into becoming a woman of the home. She told me never to eat meat before I was oriented. She also advised me never to eat mushrooms if I think I’m pregnant to avoid miscarriage…….”*

To further support the claim, another FGD participant, a pregnant woman, added,

*“……. Back in those days, my father used to tell us from ‘wang oo’ never to eat chicken when I’m married. He added that it was disrespectful for a woman to eat chicken. This got stacked in mind and up to now I still do not eat chicken….”*

### Individual characteristics

Some taboos are linked to spiritual attachment and/or appeasement. It was vividly expressed by many of the participants that some clans in Acholi have ‘*jok*’ (the spirit) that they respect. The ‘*jok*’ can befall any member of the clan. Usually, the spirit demands avoidance of certain foods, and failure to adhere to the wish of the spirit, the individual could face severe consequences. One KII participant, a TBA, expounded,

*“……. The ‘jok koma’ (my spirit) does not allow me to eat any four-legged animals. When I eat it, I get paralyzed and develop sores all over my body. This started when I was still young and, in my life, I haven’t eaten any meat from animals with four legs. There was a day I accidentally ate beef from a wedding ceremony, I almost died, I completely became paralyzed, and people could not explain what was happening to me….”*

### Social dimensions

From the views expressed, certain taboos seem to be losing relevance, but societal pressure and influence appear to play a large role in holding people onto it. Society often expects adherence to preset norms without questioning. There were general concerns that elders, particularly older women, usually impose and/or dictate certain taboos on younger women. Adherence to some taboos was thus linked to fear of reprimand and rejection. One of the FGD participants, a pregnant woman, expressed,

*“…….My mother-in-law told me his son would not take cows to our home if I indulged in eating meat. I asked myself how eating meat was related to my marriage…….”*

Another KII participant, a TBA, added,

*“………When I was a girl, my mother told me never to eat chicken if I wanted to be a respectful girl. She gave me an example of a girl in the neighborhood who was chased from her marital home for eating the gizzard of a chicken….”*

**Misbeliefs and other taboos during pregnancy in the Acholi community.**

Respondents were further asked about their awareness regarding other taboos or misconceptions attached to pregnancy. Most of the KII and FGD participants expressed varied views pertaining to taboos that pregnant women should respect. A total of 9 issues were raised during interviews with key informants and discussions with the focus group participants (Table 3).

**Table 3:** Summary of other taboos/misbeliefs during pregnancy and reasons attached to them, Acholi subregion, Northern Uganda, 2022

Misbeliefs/other taboos	Reasons attached to it	Participants who mentioned the misbelief/taboo and reasons
Anybody who killed a bird should not enter a house where a pregnant woman is lives	Causes fetal blindness	A few of the pregnant women
Pregnant women should not touch soil from graves	Causes miscarriage	A few of the pregnant women
Pregnant women are not allowed to shape or shave their hair (it makes the baby’s head bald)	It makes the baby’s head becomes bald	A few of the pregnant women
Pregnant women should not walk over anthills	It causes miscarriage, body and sore tongue	Majority of the TBAS, Spouses of the pregnant women and a few of the pregnant women
Pregnant women should not slaughter chicken or touch slaughtered chicken	It causes miscariage	Majority of the TBAs and pregnant women
Pregnant women should not have sex especially in the 3rd trimester	Causes the sperm to plaster onto the fetal body	Majority of the Pregnant women and their spouses
Pregnant women are not allowed to sit on animal’s hide or skin	Causes miscarriage	A few of the TBAs and Rwodi
Pregnant women are not allowed to touch needle	Causes miscarriage	Majority of the TBAs, Pregnant women and Rwodi

Soil from graves denotes the soil particles piled out when digging out burial graves

Five out of nine taboos mentioned were linked to miscarriage. KII and FGD participants expressed that most of the Acholi taboos, especially those practiced during pregnancy, aim to protect the fetus. In support of these views, one of the Rwodi of Ker Kal Kwaro Acholi stated,

*“……. in our culture, taboos during pregnancy are meant to protect the fetus from coming out preterm. People tend to adhere more to taboos than formal laws because of the immediate consequences associated with taboos. So we use taboos to shape behaviors that would harm lives and societies….”*

Although in some clans these beliefs and taboos are still strongly held, the majority of the respondents think that the adherence to such things is waning in most Acholi communities. An FGD participant, a pregnant mother, and a KI respondent put it straight that,

*“……. These things called taboos no longer work. Women held needles, sat on hides or skins, and ran over anthills, but nothing happened to them. We need to find a way of detaching ourselves from such attachments…….”*

However, one of the spouses of a pregnant woman indicated,

*“……It’s unfortunate that today’s generation is losing track of their culture. Taboo in some clans is alive, let no one deceive you that these things are outdated. A case in point, in the neighboring clan last year, a pregnant woman in the fourth month of pregnancy had a miscarriage because she walked over an anthill when she went to fetch firewood. It truly depends on the family one comes from…….”*

## Discussion

This study explored misbeliefs and food taboos during pregnancy and early infancy among rural Acholi communities in Northern Uganda. From the results, participants aged 36 years and above were richer in information regarding food taboos than their younger counterparts. Younger people, especially those younger than 18 years, felt slightly uncomfortable talking about the topic even when the researcher could see the responses on their faces. The researcher was able to probe and make the discussions more interactive and participatory to enable them to bring out the issues clearly. In Acholi, it is forbidden to talk about taboos in public, especially by young people, which could explain why younger respondents felt uneasy discussing taboos. Their role is simply to adhere to it. The information gap about food taboos among young people could also be due to the breakdown in the informal teaching system, the ‘*Wang oo’*. One of the Rwodi of Ker Kal Kwaro Acholi indicated that most young people are losing direction and are becoming ignorant of their culture because the ‘*Wang oo’*, which used to be a sort of informal school, is no longer common. Fournier [27] reported that the 20-year civil war in Acholi injured the formal space to bind the community. The cessation of the ‘*Wang oo’* (a central, communal fireplace where elements of the culture – stories, songs, riddles, and parables – were told and passed) severely undermined the cultural systems and the continuity of traditional values from one generation to the next [28, 29]. Additionally, the advent of Christianity and formal education, as explained by one of the respondents, distanced children even more from older people, who could be a rich source of information.

Among the Acholi community, food taboos seem to target women more and men to a lesser extent. Most of the taboos mentioned other than those practiced during pregnancy excluded men. It was eminent during the discussion that women had more information about food taboos than men, and the practices appeared to be more prevalent among the women. In several instances, the researcher witnessed men consulting their wives for certain responses. Lambek [30] found that women bear heavier burdens of taboos and observance than men. He added that the gender bias of taboos raises a complex of issues related to the paradoxical relations between autonomy and values that were beyond the scope of his study. This finding agrees with the results of this study.

The results from the present study show medical conditions, cultural beliefs, level of income, availability of food items, prescribed menu, individual food preference, state of pregnancy, and knowledge about food and nutrition as key factors that influence maternal nutrition during pregnancy. During pregnancy, physiological changes that occur due to hormonal interactions, excessive weight gain, fat disposition, and modification in cardiovascular, respiratory, and gastrointestinal functions can affect the woman’s dietary intake [31, 32]. The changes in diet during pregnancy appear to reflect a woman’s efforts to balance physiological changes accompanying pregnancy with the desire for a healthy baby. Forbes, Graham [33] found that women frequently select and accept certain foods for the health of their babies and to satisfy cravings. [34, 35] reported that the interaction of individual values and beliefs about nutrition in pregnancy, nutrition knowledge, physical and physiological changes with the determinants of other eating behaviors often change the woman’s food choices during pregnancy. Further compounding the results from the present study, Versele, Stok [36] found social influence, home/environment food availability, food knowledge, and self-regulation to influence food choices during pregnancy.

This study summarizes the foods considered special by the respondents into four main categories, namely, carbohydrate sources (which include porridge, rice, cassava, and maize), protein sources (e.g., pulses, fish, meat, nuts legumes), fat and oil (such as nuts, ‘*odii*’ peanut butter, and sheer butter), vitamins, and minerals (mainly from fruits and vegetables). Other components of the special nutritional requirement during pregnancy cited in this study are oral micronutrient supplementation, especially iron and folic acid. For infants younger than 6 months, the results show that breast milk is preferred, although other respondents indicated feeding infants older than two months on sugar solution, soup, and porridge as alternative meals, especially for mothers with breastfeeding challenges. According to Trumbo, Schlicker [37], and the Academy of Nutrition and Dietetics, approximately 300 extra calories are needed each day to maintain a healthy pregnancy. These calories should come from a balanced diet of proteins, fruits, vegetables, and whole grains. Protein intake during pregnancy is 60 g/day, which is equivalent to 1.1 g consumption of protein/kg/day [37]. Meyers, Hellwig [38] recommend that women take folic acid from fortified food or supplements daily in addition to consuming a diet rich in food sources of folate to reduce the risk for neural tube defects in their offspring. Accordingly, breastfeeding and breast milk are the global standards for infant feeding. Experts advise that children should be breastfed exclusively for the first 6 months, and this should continue for at least through the first year of life [39]. Although the foods listed as special for women to consume during pregnancy in this study agree with some of these recommendations and other available feeding guidelines [38–41], the knowledge regarding the consumption of these foods is unclear and is beyond the scope of this present study. However, efforts to promote adherence to culturally appropriate nutrition practices and education may be an important care practice for many pregnant women.

This study found more than fourteen foods, namely, Offals, Chicken, Wild birds, Smoked meat and fish, Sugarcane, Garden egg (‘Tula’), Groundnut, Bush meat, Mushrooms, Honey, Sour fruits or meals (oranges, mango, passion fruits, lemon tamarind and ‘Malakwang’), Goat’s meat, *‘Lalaa’* (the bitter green leafy vegetable), and ‘*Lamola*’ (*Hyptis spicigera*), also known as the black sesame, that are held as taboos among the Acholi community. The consumption of these foods is prohibited during pregnancy for pertinent reasons cited by the study participants. The main reasons advanced for the ban on these foods held as taboo in this study were the fear that it would cause the umbilical cord to tie around the neck of the fetus and thus choke it, leading to miscarriage, causing blindness to the fetus, causing navel/umbilical cord infection, resulting in generalized fetal skin fissure, skin inflammation to the fetus, causing whitish plasters onto the fetal baby, leading to infertility in women, causing fetal skin rashes, leading to large babies, erectile dysfunction, and respect for the men. It is important to note that the practices of these food taboos are not cast in stone throughout the Acholi community but rather specific to some clans. Each clan has its own clearly distinct cultural norms that its members uphold. Although some sections of the respondents indicated that allegiance to taboos is losing grip in the modern Acholi, many believe that the practice is still strongly held. Consistent with these findings, Tsegaye, Tamiru [42] reported instances of beliefs and taboos that restrict the consumption of certain foods during pregnancy among the rural communities of the Illu Aba Bor zone in Southwest Ethiopia. In their study, they found that food items held as taboo were vegetables (mainly cabbage and pumpkin), milk and milk products, sugarcane, fruits (e.g., bananas and avocado), and eggs. Two key reasons congruent with this result were the beliefs that the foods would plaster onto the foetal head or make the baby too large to be delivered. Similarly, Zerfu, Umeta [18] disclosed the practice of food taboos in Arsi, rural central Ethiopia, where common taboos were related to restrictions on the consumption of green leafy vegetables, yogurt, cheese, sugar cane, and green pepper during pregnancy. Furthermore, a study in the Eastern Cape, South Africa, found that meat products, fish, potatoes, fruits, beans, eggs, butternut, and pumpkin were foods commonly avoided by pregnant women [43]. In all circumstances, the reasons underlying adherence to food taboos were profoundly embedded in the individual characteristics, the influence of the social environment, and the cultural decree [19, 42]. As revealed in the present study, individual characteristics such as possession of some spirits may limit them from consuming certain foods. Compatible with this finding, the ‘*trumba*’ spirit of the Malagasy speakers prohibited the host from eating chicken. This is an example to show that taboos are imposed on their human host by spirits [42]. It is evident that most of the foods reported as taboo in the present and previous studies provide rich sources of essential macro- and micronutrients, which are vital for maternal health, foetal development, and desired pregnancy outcomes, including infant and child growth and development. For example, fruits and vegetables supply dozens of essential nutrients, including potassium, magnesium, dietary fibre, folate, and vitamins A and C; fruits and a variety of other bioactive substances that play a key role in health [44]. Although there is no plausible proof yet to support the suggestion that the practices of food taboos during pregnancy could be the reason for the poor nutrition outcomes in the Acholi subregion, this study set forth a novel understanding of the complexities surrounding maternal and child nutrition in the region, particularly from the cultural perspective. Further study is recommended to investigate the cause‒effect relationship between food taboos and nutritional outcomes in the Acholi.

The results from this study also revealed that food taboos during pregnancy are not practiced in isolation but in association with other established sets of cultural norms. Depending on the clan, the Acholi people attach several taboos to pregnancy. In some families, as seen in our results, a pregnant woman is not allowed to associate herself with certain practices. Reasons in support of these restrictions were mainly related to the avoidance of miscarriage. With respect to the avoidance of sex during pregnancy, the justification was the perception that the sperm would plaster onto the foetal body and therefore look unpleasant during delivery. Although participants expressed the realities of consequences associated with defiance, there is no scientific evidence to explain the phenomena. Contrary to the view attached to sexual intercourse in this study, Shojaa, Jouybari [45] reported the reasons to evade sex during pregnancy among Iranian women to be associated with the fear of abortion, fetal suffocation, fetal abnormality, rupture of fetus hymen, and concern of harm to the mother. Orji, Ogunlola [46] also found the reasons for the avoidance of sex during pregnancy among pregnant women in Ilesa, Nigeria, to include nausea and vomiting during early pregnancy, fear of miscarriage, fear of harming the fetus, physical awkwardness, lack of interest, discomfort, fear of membrane rupture, fear of infection, and fatigue. It appears that perceptions of sexual intercourse during pregnancy as well as the nature of other beliefs and practices vary with cultural and social contexts. A study in the District of Ilocos Sur, Philippines, Oyo State, Nigeria, and Shama District of the Western Region, Ghana, reported disparate taboos and misconceptions and reasons attached to them [47–49].

The main strength of this current study is its ability to document for the first time an account of taboos, especially during pregnancy in Acholi. In addition, the novelty finding regarding the adherence to culturally appropriate nutrition practices and education may offer an important care practice for many pregnant women especially those living in remote settings for improved nutrition outcomes. However, this study was only limited to exploring the taboos and cultural misbeliefs associated with nutrition during pregnancy, but not the cause-effect relationship. Future studies should examine this linkage. Another limitation pertinent to this study was the young women being hesitant to openly talk about taboos during the FGDs because of fear of retribution. The researcher addressed this by categorising the FGD participants into different age groups. Each age group was interviewed separately to ensure a free expression of views during the discussions.

## Conclusion

This study unveiled the eminent food taboos, other cultural beliefs, and misbeliefs related to maternal and infant nutrition and general well-being during pregnancy within the Acholi community in Northern Uganda. Adherence to these practices was found to be driven by established cultural edicts, social contexts, and individual characteristics that vary from clan to clan. Grossly, the anxiety of losing the fetus, fear of poor birth outcomes, the suspicion that the mother would die or become impotent, and the worry of a social outcast were put forward as key reasons for the assent to these taboos. Notably, the foods condemned for consumption during gestation mainly include offals, chicken, wild birds, smoked meat, smoked fish, sugarcane, garden egg (‘*Tula*’), groundnuts, wild meat, mushrooms, honey, ‘*Malakwang’*, oranges, mango, passion fruits, lemon, tamarind, goat meat, ‘*Lalaa*’ (the bitter green leafy vegetable), ‘*Lamola*’, and black sesame (*Hyptis spicigera*). From an expert point of view and the global standards on maternal nutrition, restricting the consumption of these vital foods during the critical period of developmental programming would likely leave dire implications on the development of the growing fetus and maternal health, including child growth and development later in life. From the results, health care providers appear to pay less attention to issues of taboos during their routine encounters with pregnant women. Based on the reality expressed regarding these practices, nutritional counselling and education should focus more on addressing food taboos. Appropriate nutrition interventions targeting pregnant women and their spouses, school-going children, adolescent girls, and cultural leaders in their respective points of contact would go a long way to confronting this barrier for improved nutrition outcomes in the region. Further studies focusing on investigating the cause-effect relationship between food taboos and specific nutrition outcomes are recommended.

### Electronic supplementary material

Below is the link to the electronic supplementary material.

Supplementary Material 1

## Data Availability

The datasets used and/or analysed during the current study are available from the corresponding author on reasonable request.

## Abbreviations

ANC: Antenatal care
FGD: Focus Group Discussion
HC: Health Centre
HCP: Health Care Provider
IDI: In-depth Interview
KI: Key Investigators
KII: Key Informant Interview
MIYCAN: Maternal Infant Young Child and Adolescent Nutrition
PI: Principal Investigator
TBA: Traditional Birth Attendant
UBOS: Uganda Bureau of Statistics
UDHS: Uganda Demographic and Health Survey
UNAP: Uganda Nutrition Action Plan
UNICEF: United Nations Children’s Fund
VHT: Village Health Team
