# Supplementary material for: The misbeliefs and food taboos during pregnancy and early infancy: a pitfall to attaining adequate maternal and child nutrition outcomes among the rural Acholi communities in Northern Uganda
Source: BMC Nutr. 2023 Nov 6;9:126. doi: 10.1186/s40795-023-00789-8 (PMC10629057; doi:10.1186/s40795-023-00789-8)
Supplement: Supplementary file 1 — Supplementary Material 1 [file 40795_2023_789_MOESM1_ESM.docx]

**Supplementary file 1. FGD and KII interview guides for the research participants.**

1. **FGD interview guide for PREGNANT WOMEN**
2. What are the major foods consumed in this area?
3. What are the main factors which determine the choice of foods eaten in this community?
4. Are there any special foods for pregnant women? If yes, which ones are they?
5. When are the above foods consumed? Before, during pregnancy, or immediately after delivery?
6. Are there any special foods given to the newborn other than breastmilk?
7. Are there any food taboos in this community?

Probe: During pregnancy and after childbirth?

1. What type of food? (List them) and probe further:
2. Do you have any idea why women should adhere to food taboos during pregnancy?
3. What do you know will happen if they are consumed? Probe: Reasons attached to the consumption of the food considered taboo (Record for each food should be highlighted)
4. Do they eat these foods outside of pregnancy?

Probe: Before/After birth?

1. Do you have any experience that you are aware of that women go through when they don’t observe food taboos during pregnancy?
2. How did you know about food taboos during pregnancy? Probe:
3. How long have you known about Food Taboo in this community?

Other than Food taboos, are there other taboos and beliefs that women must observe during pregnancy? Probe further if any: What are they?

1. Other than food taboos, are there any fallacies/misbeliefs/misconceptions/illusions attached to food or feeding during pregnancy and or immediately after childbirth? Probe What they are.
2. What is the present level of adherence to food taboos in this community? Probe: Explain.
3. To the best of your knowledge, do all pregnant women in Acholi still observe Food?
4. **FGD interview guide for HUSBANDS**
5. What are the major foods consumed in this area?
6. What are the main factors which determine the choice of foods eaten in this community?
7. Are there any special foods for pregnant women? If yes, which ones are they?
8. When are the above foods consumed? Before, during pregnancy, or immediately after delivery?
9. Are there any special foods given to the newborn other than breastmilk?
10. Are there any food taboos in this community?

Probe: During pregnancy and after childbirth?

1. What type of food? (List them) and probe further:
2. Do you have any idea why women should adhere to food taboos during pregnancy?
3. What do you know will happen if they are consumed? Probe: Reasons attached to the consumption of the food considered taboo (Record for each food should be highlighted)
4. Do they eat these foods outside of pregnancy?

Probe: Before/After birth?

1. Do you have any experience that you are aware of that women go through when they don’t observe food taboos during pregnancy?
2. How did you know about food taboos during pregnancy? Probe:
3. How long have you known about Food Taboo in this community?

Other than Food taboos, are there other taboos and beliefs that women must observe during pregnancy? Probe further if any: What are they?

1. Other than food taboos, are there any fallacies/misbeliefs/misconceptions/illusions attached to food or feeding during pregnancy and or immediately after childbirth? Probe What they are.
2. What is the present level of adherence to food taboos in this community? Probe: Explain.
3. To the best of your knowledge, do all pregnant women in Acholi still observe Food?
4. **KII guide for Health Care Providers and VHTs**
5. What are the major foods consumed in this area?
6. What are the main factors which determine the choice of foods eaten in this community?
7. Are there any special foods for pregnant women? If yes, which ones are they?
8. When are the above foods consumed? Before, during pregnancy, or immediately after delivery?
9. Are there any special foods given to the newborn other than breastmilk?
10. Are there any food taboos in this community?

Probe: During pregnancy and after childbirth?

1. What type of food? (List them) and probe further:
2. Do you have any idea why women should adhere to food taboos during pregnancy?
3. What do you know will happen if they are consumed? Probe: Reasons attached to the consumption of the food considered taboo (Record for each food should be highlighted)
4. Do they eat these foods outside of pregnancy?

Probe: Before/After birth?

1. Do you have any experience that you are aware of that women go through when they don’t observe food taboos during pregnancy?
2. How did you know about food taboos during pregnancy? Probe:
3. How long have you known about Food Taboo in this community?

Other than Food taboos, are there other taboos and beliefs that women must observe during pregnancy? Probe further if any: What are they?

1. Other than food taboos, are there any fallacies/misbeliefs/misconceptions/illusions attached to food or feeding during pregnancy and or immediately after childbirth? Probe What they are.
2. What is the present level of adherence to food taboos in this community? Probe: Explain.
3. To the best of your knowledge, do all pregnant women in Acholi still observe Food?
4. What influence do you think misbeliefs and or food taboos practice have on maternal and child nutrition, particularly during pregnancy and immediately after birth?

Probe further if any: What are they?

1. **KII study guide for RWODI and TBAs**
2. What are the major foods consumed in this area?
3. What are the main factors which determine the choice of foods eaten in this community?
4. Are there any special foods for pregnant women? If yes, which ones are they?
5. When are the above foods consumed? Before, during pregnancy, or immediately after delivery?
6. Are there any special foods given to the newborn other than breastmilk?
7. Are there any food taboos in this community?

Probe: During pregnancy and after childbirth?

1. What type of food? (List them) and probe further:
2. Do you have any idea why women should adhere to food taboos during pregnancy?
3. What do you know will happen if they are consumed? Probe: Reasons attached to the consumption of the food considered taboo (Record for each food should be highlighted)
4. Do they eat these foods outside of pregnancy?

Probe: Before/After birth?

1. Do you have any experience that you are aware of that women go through when they don’t observe food taboos during pregnancy?
2. How did you know about food taboos during pregnancy? Probe:
3. How long have you known about Food Taboo in this community?

Other than Food taboos, are there other taboos and beliefs that women must observe during pregnancy? Probe further if any: What are they?

1. Other than food taboos, are there any fallacies/misbeliefs/misconceptions/illusions attached to food or feeding during pregnancy and or immediately after childbirth? Probe What they are.
2. What is the present level of adherence to food taboos in this community? Probe: Explain.
3. To the best of your knowledge, do all pregnant women in Acholi still observe Food?
